# Comparison of outcomes with and without intrastent placement during PMS surgery

**DOI:** 10.1038/s41598-025-87259-2

**Published:** 2025-01-23

**Authors:** Yusaku Miura, Ken Fukuda, Kenji Yamashiro

**Affiliations:** Department of Ophthalmology and Visual Science, Kochi Medical School, Kochi University, Kohasu, Oko-cho, Nankoku City, 783-8505 Kochi Japan

**Keywords:** Glaucoma, PreserFlo microShunt, Postoperative hypotony, Glaucoma, Ocular hypertension

## Abstract

To assess the efficacy of using a nylon suture as a stent in the PreserFlo MicroShunt (PMS) lumen to prevent postoperative hypotony, 59 eyes that underwent PMS implantation with follow-up for > 6 months were analyzed. Patients were divided into no intrastenting (NST) and intrastenting (ST) groups, with the ST group subdivided into 9 − 0 nylon suture fully placed (9 F), 9 − 0 nylon suture placement in only half of the lumen (9 H), 10 − 0 nylon suture fully placed (10 F), and 10 − 0 nylon suture placement in only half of the lumen (10 H). The distribution was as follows: 23 eyes in the NST group, 10 in the 9 F group, 9 in the 9 H group, 11 in the 10 F group, and 6 in the 10 H group. No significant differences were observed in preoperative and 6-month postoperative intraocular pressure, number of glaucoma medications, or cumulative survival rate between groups. Postoperative hypotony occurred in 13 eyes (56.5%) in the NST group, one (2.78%) in the ST group (*p* = 0.00014). Post-intrastent removal, hypotony occurred in 6 eyes (16.7%) in the ST group. These findings suggest that intrastent placement effectively prevents postoperative hypotony, regardless of nylon suture diameter or insertion length; however, timing is crucial as hypotony may occur after removal.

## Introduction

**Fig. 1:**
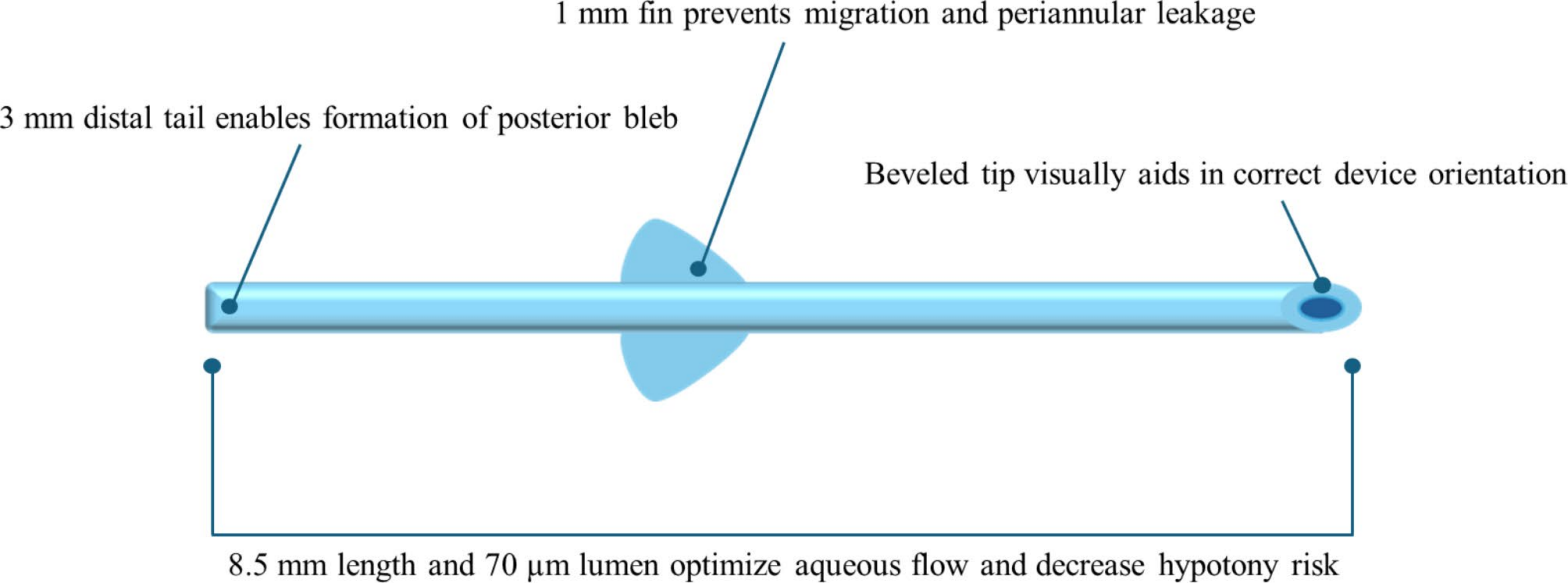
Illustration of the Preserflo MicroShunt.

The PreserFlo MicroShunt (PMS; Santen Pharmaceutical Co., Ltd., Osaka, Japan) was placed ab externo in the anterior chamber to drain the aqueous humor from the anterior chamber into the subtenon/subconjunctival space to form a conjunctival bleb, thereby reducing intraocular pressure (IOP)^1^. Unlike TLE, PMS does not require postoperative laser suture lysis, is a simple procedure, and is associated with fewer postoperative complications and procedures^2^. Although the lumen diameter and total length of the PMS are designed to minimize the incidence of postoperative hypotony based on the Hagen-Poiseuille equation (Fig. 1), the frequency of hypotony after PMS implantation reportedly ranges from 11.1 to 69%, and hypotony is the most common postoperative complication of PMS^2–8^. Hypotony can lead to serious complications, such as choroidal detachment, choroidal hemorrhage, visual loss due to hypotony maculopathy, and corneal endothelial damage due to the shallow anterior chamber. Therefore, preoperative precautions should be taken to prevent postoperative hypotony.

Previous studies have explored methods to prevent postoperative hypotony using 10 − 0 nylon sutures placed over the entire length of the PMS lumen^9^ or 8 − 0 beveled polyamide monofil sutures placed in approximately 1/3 of the entire length of the PMS lumen^10^. These techniques aim to limit the amount of aqueous humor filtration from the PMS lumen. Although intrastent placement in the PMS lumen may be effective in preventing postoperative hypotony, optimal stent diameter and length of placement have not yet been established. In the present study, we evaluated the efficacy of 9 − 0 and 10 − 0 nylon sutures in patients treated with full-length or half-length stents.

## Materials and methods

All patients who underwent PMS implantation by a glaucoma surgeon (YM) at Kochi University Hospital between June 2023 and November 2023 and were followed up 6 months postoperatively were retrospectively studied. Clinical data were collected according to the principles of the Declaration of Helsinki and approved by the clinical research committee of the hospital. Informed consent was obtained from all participants before surgery. Preoperatively, patients underwent slit-lamp examination, fundus examination, IOP measurement with a Goldman applanation tonometer (Haag-Streit, Koeniz, Switzerland), best-corrected visual acuity (BCVA), and Humphrey Visual Field 30 − 2. Data regarding age, sex, number of glaucoma medications, and previous ophthalmic surgeries were also collected. Postoperative data, including slit-lamp examinations, IOP measurements, number of glaucoma medications, complications, and interventions, were collected on day 1, week 1, and months 1, 2, 3, and 6. The patients were divided into two groups: no intrastent (NST) and intrastent (ST). In addition, the ST group was further divided into four groups: 9 − 0 nylon suture fully placed in the lumen (9 F), 9 − 0 nylon suture placed in only half of the lumen (9 H), 10 − 0 nylon suture fully placed in the lumen (10 F), and 10 − 0 nylon suture placed in only half of the lumen (10 H).

### Surgical procedure

**Fig. 2:**
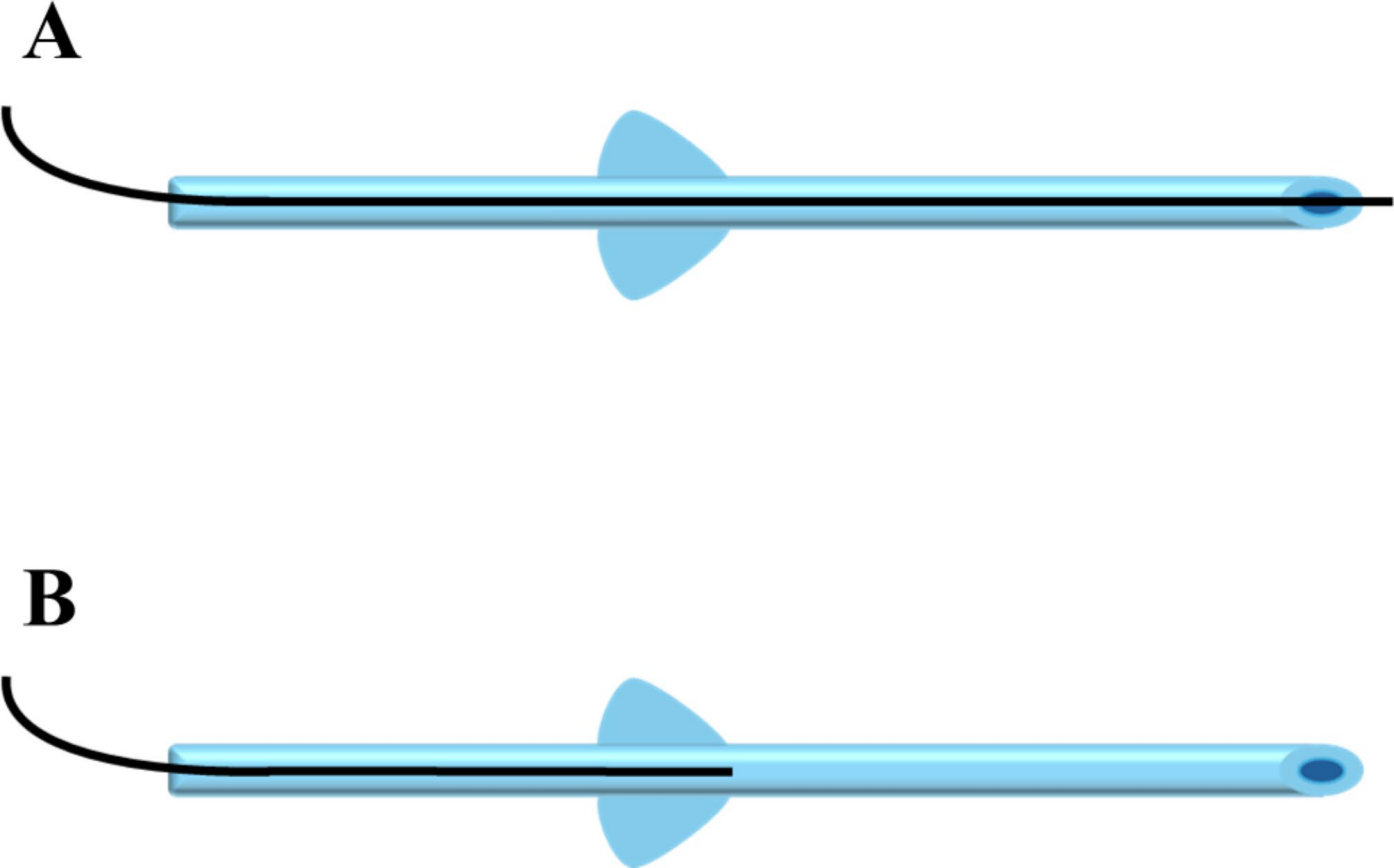
intrastent placement. (**A**): Intrastent placement in the full length of the lumen. (**B**): Intrastent placement in only half of the lumen.

**Fig. 3:**
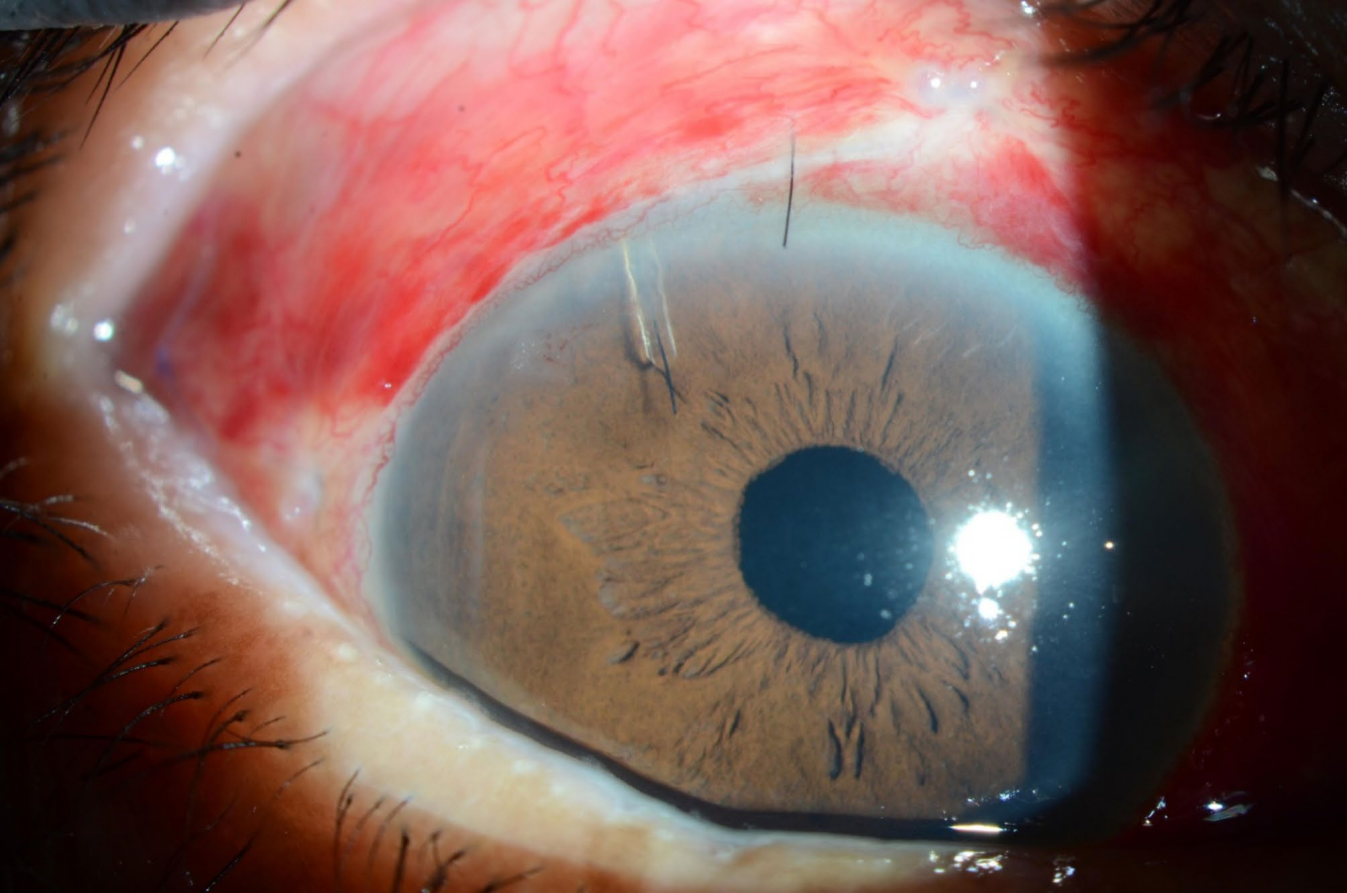
Slit-lamp photograph of the intrastent placement in the full length of the lumen.

PMS implantation was performed according to a previously published recommendation^4^. Briefly, conjunctival peritomy was performed under sub-Tenon’s sac anesthesia, and sponges soaked in mitomycin C (0.4 mg/mL) were placed into the subconjunctival space for 4 min. A scleral tunnel and a pocket were created 3 mm from the limbus using a double-step knife. The PMS was inserted into the scleral tunnel, the PMS fin was fixed in the scleral pocket, and aqueous outflow from the external end of the PMS was checked. In groups 9 F and 10 F, a 9 − 0 or 10 − 0 nylon suture was inserted as a intrastent through the lumen of the outer end of the PMS and advanced to the inner end. The intrastent was externalized through the sclera onto the conjunctiva without placing a knot at the distal end to allow later removal. Similarly, in groups 9 H and 10 H, a 9 − 0 or 10 − 0 nylon sutures was inserted through the lumen of the outer end of the PMS and advanced to a position beyond the fin such that it was approximately half of the total length of the PMS (Figs. 2 and 3). Finally, the conjunctiva was sutured to the limbus with an 8 − 0 Vicryl suture, and 0.8 mL of betamethasone (2 mg) was administered via subconjunctival injection. Postoperatively, all patients were instructed to discontinue their previous glaucoma medications.

After local anesthetic eye drops were administered, the end of the intrastent on the conjunctiva was grasped and pulled out of the PMS lumen under a slit lamp for intrastent removal. Basically, the criterion for intrastent removal was an IOP ≥ 15 mmHg. However, even if the IOP was between 12 and 15 mmHg, the intrastent was removed at the glaucoma surgeon (Y.M.)’s discretion if necessary, considering the target IOP, bleb formation, and individual risk factors.

### Statistical analysis

We defined hypotony as an IOP < 6 mmHg during any postoperative visit. BCVA was converted to the logarithm of the minimum angle of resolution (logMAR) units for statistical analysis. All continuous data are expressed as the mean ± standard deviation. The Wilcoxon signed-rank test was used to compare preoperative and last-visit measurements of IOP and the number of glaucoma medications. Surgical success was defined as: (1) IOP ≤ 21, and a reduction by more than 20% with or without glaucoma medications; (2) no loss of light perception; (3) no additional glaucoma surgery. Transient IOP elevation within one month postoperatively was considered an IOP spike and was not classified as surgical failure. Differences in survival rates among groups were compared using the log-rank test. The Kruskal–Wallis and Steel tests were used to evaluate group differences between continuous variables. The incidence of complications was compared between groups using Fisher’s exact test. Statistical significance was set at *p* < 0.05. All data were entered into an Excel spreadsheet (Microsoft Corp., Redmond, WA, USA) and analyzed using Excel 2016 with the add-in software Statcel 4.

## Results

PMS was implanted in 59 eyes of 44 patients with glaucoma. Of these, 23 eyes were in the NST group, 10 were in the 9 F group, 9 were in the 9 H group, 11 were in the 10 F group, and 6 were in the 10 H group. The mean age was 68.7 years in the NST group, 76.2 years in the 9 F group, 75.0 years in the 9 H group, 77.9 years in the 10 F group, and 75.5 years in the 10 H group. Baseline parameters did not differ significantly between the groups (Table 1).

**Table 1:** Baseline demographics and clinical characteristics.

	NST	9 F	9 H	10 F	10 H	*P* value
No. of patients (no. of eyes)	16 (23)	10 (10)	8 (9)	10 (11)	6 (6)	
Age (years)	68.7	76.2	75.0	77.9	75.5	0.11
Sex (M/F)	9/14	5/5	3/6	7/4	4/2	0.49
Eye (R/L)	9/14	5/5	6/3	6/5	3/3	0.15
Glaucoma diagnosis, n(%)						
Primary open-angle glaucoma	19 (82.6)	5 (50.0)	6 (66,7)	9 (81.8)	4 (66.7)	0.34
Pseudoexfoliation glaucoma	4 (17.4)	5 (50.0)	3 (33.3)	2 (18.2)	2 (33.3)	
Previous glaucoma surgery, n(%)						
Trabeculotomy	7 (30.4)	–	–	2 (18.2)	1 (16.7)	0.14
Trabeculectomy	4 (17.4)	1 (10.0)	–	1 (9.1)	1 (16.7)	0.71
Ahmed glaucoma valve	–	1 (10.0)	2 (22.2)	1 (9.1)	–	0.21
Preoperative visual fields–mean deviation (dB, mean ± SD)	–15.2	–21.9	–17.7	–19.0	–22.9	0.37
Combination with cataract surgery(%)	7 (30.4)	5 (50.0)	4 (44.4)	3 (27.3)	5 (83.3)	0.15

As shown in Table 2, preoperative IOP was not significantly different among the five groups (*p* = 0.33): 21.0 ± 5.8 mmHg in the NST group, 18.9 ± 3.0 mmHg in the 9 F group, 17.8 ± 4.0 mmHg in the 9 H group, 18.4 ± 3.8 mmHg in the 10 F group, and 17.0 ± 3.3 mmHg in the 10 H group, respectively. The IOP decreased on postoperative day 1:7.78 ± 7.1 mmHg in the NST group, 17.6 ± 8.8 mmHg in the 9 F group, 11.3 ± 3.5 mmHg in the 9 H group, 13.9 ± 5.6 mmHg in the 10 F group, and 14.8 ± 6.4 mmHg in the 10 H group. The NST group had a significantly lower IOP than the other four groups (*p* = 0.00011), whereas no significant difference in IOP was observed among the four ST groups (*p* = 0.16). Thereafter, IOP increased in the NST group, whereas IOP tended to decrease in the ST group. Although the final IOP was nominally higher in NST than the four groups of the ST group, the final IOP was not significantly different among the five groups (*p* = 0.24): 14.0 ± 4.4 mmHg in the NST group, 12.6 ± 4.8 mmHg in the 9 A group, 12.1 ± 2.0 mmHg in the 9 H group, 11.1 ± 2.4 mmHg in the 10 A group, and 10.3 ± 3.1 mmHg in the 10 H group. The final IOP at 6 months after surgery was significantly lower than that at baseline in all groups (*p* < 0.05 in each group).

**Table 2:** Comparisons in preoperative and postoperative IOP.

	NST	9 F	9 H	10 F	10 H	*P* value
Baseline	21.0 ± 5.8	18.9 ± 3.0	17.8 ± 4.0	18.4 ± 3.8	17.0 ± 3.3	0.33
After surgery						
1 day	7.78 ± 7.1	17.6 ± 8.8	11.3 ± 3.5	13.9 ± 5.6	14.8 ± 6.4	0.00011*
1 week	7.74 ± 3.8	12.3 ± 5.8	13.1 ± 8.6	9.54 ± 3.7	10.2 ± 3.9	0.057
1 month	11.7 ± 4.6	10.7 ± 5.2	11.3 ± 3.2	9.91 ± 3.1	9.67 ± 2.4	0.77
2 months	14.0 ± 5.4	11.3 ± 7.2	11.4 ± 2.7	10.3 ± 2.0	10.0 ± 1.4	0.22
3 months	13.8 ± 5.1	12.6 ± 4.8	10.8 ± 2.3	9.72 ± 2.1	10.0 ± 2.0	0.15
6 months	14.0 ± 4.4	12.6 ± 4.8	12.1 ± 2.0	11.1 ± 2.4	10.3 ± 3.1	0.24

Values are presented as IOP, mmHg.

Data are the mean ± SD.

*IOP* intraocular pressure.

* Significance level of 5%.

The preoperative number of glaucoma medications did not significantly differ among the five groups (*p* = 0.90): 3.8 ± 1.0 in the NST group, 3.6 ± 1.4 in the 9 F group, 3.8 ± 0.4 in the 9 H group, 3.8 ± 1.1 in the 10 F group, and 4.2 ± 0.8 in the 10 H group. After the surgery, the number of glaucoma medications significantly decreased at 6 months postoperatively (*p* < 0.05 in each group): 1.0 ± 1.6 in the NST group, 0.70 ± 1.5 in the 9 F group, 0.67 ± 1,4 in the 9 H group, 0 ± 0 in the 10 F group, and 0.67 ± 1.6 in the 10 H group. The postoperative number of glaucoma medications was not significantly different among the five groups (*p* = 0.31).

Preoperative BCVA was 0.22 ± 0.44 in the NST group, 0.82 ± 0.86 in the 9 F group, 0.29 ± 0.23 in the 9 H group, 0.35 ± 0.56 in the 10 F group, and 0.83 ± 0.70 in the 10 H group (*p* = 0.050). BCVA at 6 months postoperatively was 0.21 ± 0.47 in the NST group, 0.80 ± 0.89 in the 9 F group, 0.17 ± 0.26 in the 9 H group, 0.34 ± 0.57 in the 10 F group, and 0.57 ± 0.81 in the 10 H group (*p* = 0.30).

**Fig. 4:**
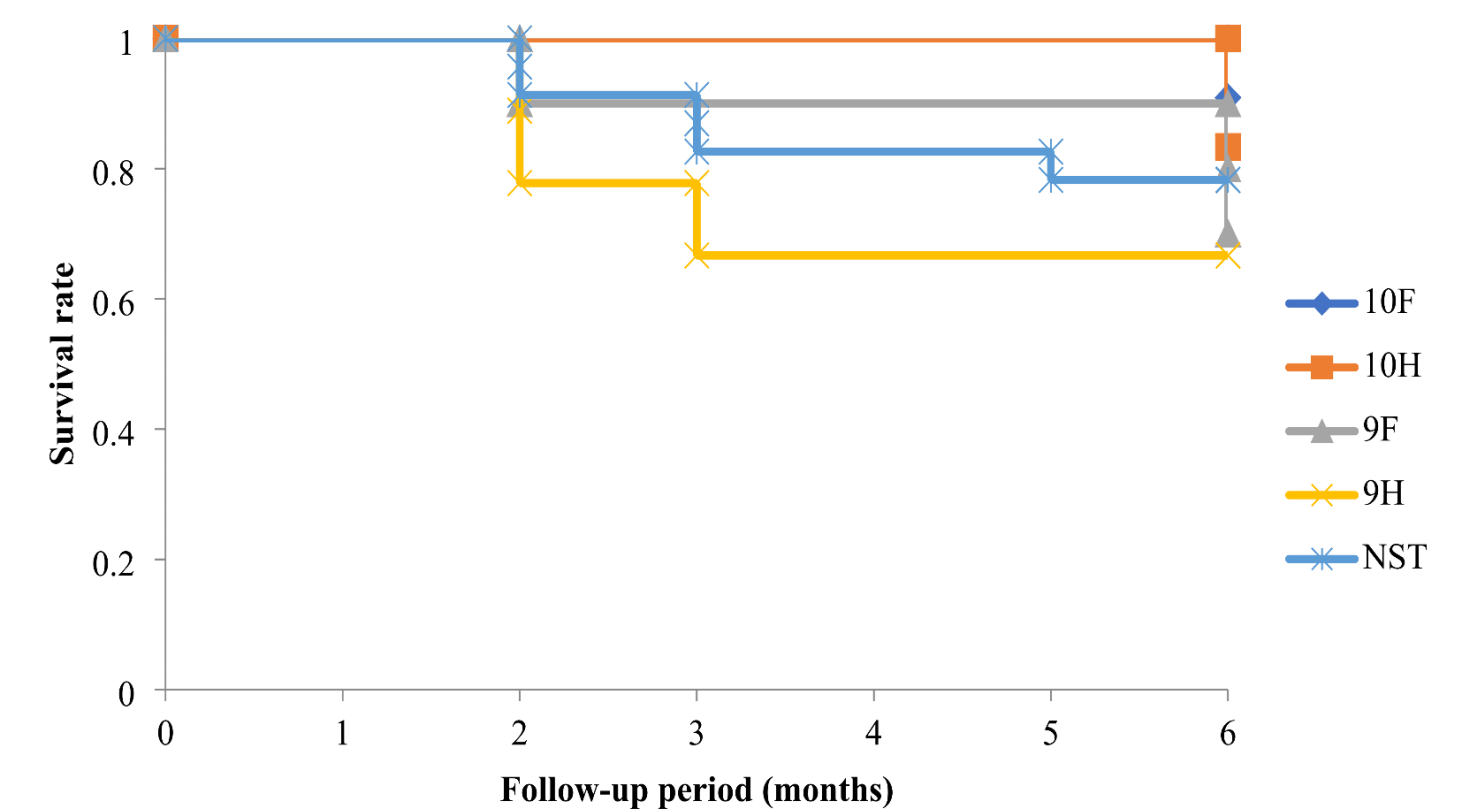
Kaplan-Meier survival curves.

Figure 4 shows the Kaplan-Meier survival curves. The success rates in the NST, 9 F, 9 H, 10 F, and 10 H groups were 78.3%, 70.0%, 66.7%, 90.9%, and 83.3%, respectively (*p* = 0.68). Table 3 shows the postoperative complications and interventions. The incidence of postoperative hypotony was significantly higher in the NST group (56.5%) than in the ST groups (2.78%) (*p* = 0.00014). Of the 13 eyes with postoperative hypotony in the NST group, four had choroidal detachment, four had hypotony maculopathy, and three had a shallow anterior chamber. Additionally, three eyes required viscoelastic material injection into the anterior chamber, and one eye needed 10 − 0 suture ab interno implantation into the inner end of the PMS lumen, similar to our previous report^11^. In contrast, hypotony in one eye in the 10 F group did not lead to any secondary complications and resolved spontaneously without treatment on the day after the onset of hypotony.

**Table 3:** Postoperative complications and interventions.

	NST	9 F	9 H	10 F	10 H	*P* value
Complications						
Hypotony	13(56.5)	0	0	1(9.1)	0	0.00014*
After intrastent removal	-	2(20.0)	1(11.1)	1(9.1)	2(33.3)	0.59
Choroidal detachment	4(17.4)	0	0	1(9.1)	1(16.7)	0.43
Hypotony maculopathy	4(17.4)	0	0	0	1(16.7)	0.23
Shallow anterior chamber	3(13.0)	0	0	0	0	0.29
Hyphema	3(13.0)	0	1(11.1)	1(9.1)	0	0.70
IOP spike (IOP > 30mmHg)	1(4.3)	1(10.0)	1(11.1)	0	0	0.71
Tube occlusion	0	0	1(11.1)	0	0	0.23
Blebitis	0	0	0	0	1(16.7)	0.061
Interventions						
Needling	5(21.7)	1(10.0)	0	0	0	0.18
AC formation with OVD	3(13.0)	0	0	1(9.1)	0	0.50
Intraluminal stent placement	1(4.3)	0	0	0	0	0.81
Choroidal tapping	0	0	0	1(9.1)	0	0.070
Tube flush	0	0	1(11.1)	0	0	0.23
PMS removal	0	0	0	0	1(16.7)	0.061
PMS reimplantation	1(4.3)	0	0	0	0	0.81
Bleb revision	2(8.7)	0	0	0	0	0.52

Values are presented as n (%).

*IOP* intraocular pressure, *AC* anterior chamber, *OVD* ophthalmic viscoelastic device *PMS* preserflo microshunt.

The intrastent was removed from all eyes in the ST group. The mean number of days for intrastent removal was 26.6 ± 55.3 days after surgery in group 9 F, 17.7 ± 10.6 days in group 9 H, 36.6 ± 47.0 days in group 10 F, and 12.7 ± 18.3 days in group 10 H (*p* = 0.15). The mean IOP before intrastent removal was 18.3 ± 3.4 mmHg in group 9 F, 16.7 ± 2.6 mmHg in group 9 H, 14.5 ± 2.1 mmHg in group 10 F, and 16.3 ± 2.6 mmHg in group 10 H, with group 9 F being significantly higher than group 10 F (*p* = 0.043). The mean IOP after intrastent t removal was 9.0 ± 3.9 mmHg in group 9 F, 10.2 ± 3.4 mmHg in group 9 H, 9.3 ± 3.5 mmHg in group 10 F, and 7.3 ± 2.0 mmHg in group 10 H (*p* = 0.34). In each group, the IOP decreased significantly after intrastent removal (*p* < 0.05 in each group) (Table 4). Among the four ST groups, hypotony after intrastent removal occurred in two eyes in group 9 F, one eye in group 9 H, one eye in group 10 F, and two eyes in group 10 H (*p* = 0.59). In these 6 eyes, intrastent removals underwent 2.3 ± 1.2 (range 1–4) days after surgery, and IOP before intrastent removal was 17.2 ± 3.0 (range 12–20) mmHg. Among the 36 eyes in the ST group, the incidence of postoperative hypotony was significantly higher in the 12 eyes in which the intrastent was removed within 4 days after surgery than in the 24 eyes in which it was removed 5 days after surgery (*p* = 0.00047). No significant difference in the incidence of postoperative hypotony was found between the 27 eyes with IOP ≥ 15 mmHg and the 9 eyes with 12 ≤ IOP < 15 mmHg before intrastent removal (*p* = 0.52).

**Table 4:** Intrastent removal.

	9 F	9 H	10 F	10 H	*P* value
The number of days for intrastent removal(days)	26.6 ± 55.3	17.7 ± 10.6	36.6 ± 47.0	12.7 ± 18.3	0.15
IOP before intrastent removal(mmHg)	18.3 ± 3.4	16.7 ± 2.6	14.5 ± 2.1	16.3 ± 2.6	0.043*
IOP after intrastent removal(mmHg)	9.0 ± 3.9	10.2 ± 3.4	9.3 ± 3.5	7.3 ± 2.0	0.34

*Significance level of 5%.

## Discussion

The mechanisms of postoperative hypotony after PMS implantation include: (1) overfiltration from the PMS; (2) leakage from the gap between the PMS and the scleral tunnel; and (3) decreased aqueous humor production. In this study, the incidence of postoperative hypotony was 2.78% in the ST group, in which nylon sutures were placed intraoperatively to increase aqueous humor outflow resistance. This rate was significantly lower than that of the NST group (56.5%). The main cause of postoperative hypotony has been suggested to be PMS overfiltration.

This study also demonstrated that intraoperative intrastent placement can prevent postoperative hypotony regardless of the diameter of the nylon suture or length of the incision. Compared with 9 − 0 nylon sutures, 10 − 0 nylon sutures are slightly more difficult to insert into the PMS lumen because of their shorter diameter and lower stiffness. In addition, full intrastent placement is more labor-intensive than half placement. Therefore, we believe that half the placement of the 9 − 0 nylon suture is the most efficient. Although our study found no significant difference in postoperative IOP among the four ST groups, full placement of the 9 − 0 nylon suture likely increased aqueous humor outflow resistance the most, resulting in higher postoperative IOP. Given that complications secondary to hypotony are likely to become serious in highly myopic or avitreous eyes, the full placement of a 9 − 0 nylon suture is recommended to prevent postoperative hypotony.

Our finding of no significant differences between the NST and ST groups in IOP, number of glaucoma medications, and cumulative survival rate at 6 months postoperatively suggests that intrastent placement in the PMS lumen may successfully reduce IOP comparable to that in the NST group without affecting bleb formation. Although intrastent placement may increase the risk of inflammation or infection because it connects the extraocular and intraocular spaces, we did not observe any intraocular inflammation or bleb infections. Previous studies have also reported the absence of inflammation or infection after intrastent placement^9,10^. Furthermore, intraoperative intrastent placement does not require any special surgical techniques and prolongs the surgical time by only a few minutes. As the outer end of the intrastent is located on the conjunctiva, it can be removed using a slit lamp, allowing for easy and minimally invasive intrastent removal.

Because intrastents were removed at the discretion of the glaucoma surgeon, the time of intrastent removal in different groups and within each group was so variable, and the SD was very high, making meaningful comparisons difficult. However, intrastent removal resulted in a significant IOP reduction regardless of the suture diameter or insertion length. However, six eyes developed hypotony after intrastent removal, and these cases manifested as choroidal detachment and hypotony maculopathy, which required treatment. Consequently, the timing of intrastent removal is critical. Unifying the timing of intrastent removal is challenging due to factors such as the target IOP, bleb formation, and residual visual field. However, our study found that patients who underwent intrastent removal within 4 days after surgery were more likely to develop hypotony; therefore, avoiding intrastent removal within this timeframe is preferable if possible.

The limitations of our study include its small sample size, nonrandomized study design, and short follow-up period. To clarify the differences in clinical outcomes according to the diameter and placement distance of nylon sutures and the timing of intrastent removal, a larger number of cases and longer follow-up periods are needed. Furthermore, randomized studies should be performed to eliminate biases, such as patient backgrounds.

In summary, intraoperative placement of a nylon suture as an intrastent in the PMS lumen may be effective in preventing hypotony after PMS implantation, regardless of the diameter and insertion length of the nylon suture. In addition, stent placement did not seem to affect IOP outcomes 6 months after surgery. Due to its low-cost, simplicity, and minimal invasiveness, intrastent placement is routinely recommended for PMS implantation. Further studies are required to determine the appropriate timing for intrastent removal to prevent potential hypotony.

## Data Availability

The datasets generated during and/or analyzed during the current study are available from the corresponding author on reasonable request.
